# Fatty Acid Profiles and Their Association With Autoimmunity, Insulin Sensitivity and β Cell Function in Latent Autoimmune Diabetes in Adults

**DOI:** 10.3389/fendo.2022.916981

**Published:** 2022-06-29

**Authors:** Huiqin Tian, Shiqi Wang, Yating Deng, Yanke Xing, Lin Zhao, Xia Zhang, Ping Zhang, Nan Liu, Benli Su

**Affiliations:** Department of Endocrinology and Metabolism, Second Hospital of Dalian Medical University, Dalian, China

**Keywords:** LADA (Latent Autoimmune Diabetes in Adults), fatty acid, lipidomics, slowly evolving autoimmune diabetes, polyunsaturated (essential) fatty acids

## Abstract

**Background:**

The pathogenesis of the progressive loss of beta cell function latent autoimmune diabetes in adults (LADA) remains still elusive. We aim to study the fatty acid (FA) profile in LADA.

**Subjects and methods:**

Data from 116 patients with diabetes and GADA and 249 diabetes controls without GADA selected by Propensity Score Matching were collected. FA was analyzed with liquid chromatography-tandem mass spectrometry analysis.

**Results:**

Principal factor analysis found component 1 explains 82.6% of total variance contained fatty acids from a mixed of lard oil, seafood, and vegetable diet, followed by diet predominantly from vegetable oil, a diet of high fat diet, and a diet of seafood diet. The FA heatmap looked clearly different among the three groups with more similar type 1 (t1dm) and LADA fatty acid profile. n-3 α-linolenic acid (ALA), n-3 long chain polyunsaturated fatty acid (n-3 LC-PUFA), such as Eicosapentaenoic Acid and Docosapentaenoic Acid, n-3/n-6 ratio and triene/tetraene ratio were higher in patients with type 2 diabetes (t2dm) compared with LADA and t1dm. Saturated FAs were lower in t2dm than t1dm and LADA. Arachidic acid and n-6 LC-PUFAs were lower in t2dm than in t1dm and LADA. The characteristics of FAs in LADA were in between of classical t1dm and t2dm. Patients were classified into 6 clusters by FA clusters. Only cluster 2, 3, 5 contained enough patients to be analyzed. Cluster 5 showed an insulin deficient phenotype containing more than 60% of patients with t1dm and LADA and only 12.8% of t2dm. Cluster 2 and 3 were similar. β cell function and glycemic control was better in cluster 3 homing 25% of t2dm. Cluster 2 held 28% of t1dm and LADA, in this cluster more than 60% of patients was t2dm. n-3 linolenic acid, n-3 LC-PUFAs, some n-6 LC-PUFAs, n-3/n-6 ratio and triene/tetraene ratio were negatively associated with GADA positivity while n-6 Arachidonic Acid was associated positively with GADA. Similar findings were found for insulin sensitivity and beta cell function.

**Conclusion:**

PUFA are associated with insulin sensitivity and beta cell function, and like other clinical features, FA profile distributed differently, but could not be used as makers to differentiate LADA from t1dm and t2dm.

**Ethics and Dissemination:**

This study has been approved by the Ethical Review Committee of Second Hospital of Dalian Medical University (approval number: 2021–005).

**Clinical Trial Registration:**

none

## Introduction

Latent autoimmune diabetes in adults (LADA) is a well-recognized subtype under immune-mediated type 1 diabetes (1), the pancreatic β cells are destructed by cellular-mediated autoimmunity with autoantibodies to insulin, to GAD (GADA), to the tyrosine phosphatases IA2 and IA2β, to zinc transporter 8 (ZnT8As) as autoimmune markers. Compared to classical type 1 diabetes (t1dm), LADA undergoes a slow progressive loss of β cells failure, resulted in a long duration of marginal insulin secretory capacity (2). LADA was defined as new subtype of “slowly evolving immune-mediated” under “hybrid forms of diabetes” (3). This type of diabetes is found more common that t1dm in China (4) with a prevalence of 5.9-9.2% in China with similar prevalence to its western counterpart (1.5-14.2%) (5–7). Although this subtype of diabetes has been noted for many years, the pathogenesis is still unclear why type 2 diabetes like patients with diabetes associated autoantibodies, such as GADA, IA2, Zn8TA, or insulin autoantibodies (IAA), who does not require insulin at onset, and can be treated with lifestyle modification and oral agents, progress to requiring insulin more rapidly than typical type 2 diabetes (t2dm).

Although it is commonly recognized as a specific subtype of autoimmune diabetes, there are no universally accepted criteria. More recently a group of international experts reached a consensus on recommendation for diagnosis and management of LADA (8). Clues to make a LADA diagnosis are suggested in patients with diabetes when he/she is (a) positive for GADA, IA2, ZnT8A, (b) older than 30 or 35 years of age, and (c) not insulin-dependent for at least 6-12 months. None of the criteria are categorical, especially the start of insulin therapy is entirely empirical. The recognition of LADA is of clinical practice priority, in that patient with LADA should be treated early with insulin, avoid use of sulfonylureas to prevent pancreatic islet β cell failure (7). It was also found that although at earlier stage the diabetic microvascular complications were less than t1dm or t2dm but increased more rapid and prevalent in later stage due to delayed insulin therapy (9).

The pathogenesis of LADA is elusive. Diabetes-associated autoantibodies are the hallmark clue to LADA in patients with t2dm (5, 10–20). Although trying to adopt more autoantibodies may be more efficient in predicting insulin requirement in patients with LADA, GADA is the most prevalent and not influenced by age of disease onset and last longer than other autoantibodies. Therefore, GADA is most often used in clinical research as well (6, 21–23). It has been also found there are two distinct groups of LADA with higher and lower titer of GADA (16). Genetic association studies showed similar risk HLA as t1dm, but with decreasing frequency (24, 25), only two markers in MHC class I polypeptide-related sequence A (MICA) have been shown to differentiate LADA from t1dm (26). Recently it was found genetic risk marker in transcription factor 7 like 2 (TCF7L2) found in t2dm was also associated with LADA (27, 28). The first genomic wide association study (GWS) reconfirmed patients with LADA shared the genetic background admix of t1dm and t2dm (29). Furthermore, this GWS found a new metabolic genetic locus (BFKFB3) that has been previously found associated with other autoimmune disease was also associated with LADA for the first time (29). Innate immunity studies in patients with LADA has shown inflammatory activity fell in between t1dm and t2dm (30–34).

Polyunsaturated fatty acids (PUFA) have important roles in human biology, such as controlling inflammation, being components of the cell membranes, acting as signaling molecules, and regulating gene expression (35, 36). Since the body cannot make n-3 and n-6 fatty acids on its own, their intake must be ensured through diet (37, 38). Thus, fatty acids such as α-linolenic acid (ALA C18:3 n-3) and linoleic acid (LIA; C18:2 n-6) can only be obtained from dietary sources and are therefore known as essential fatty acids (EFA). ALA and LIA undergo series of steps involving desaturation and elongation processes to form eicosanoids and compounds by elongases and desaturases. The products are long-chain-PUFAs (LC-PUFAs): dihomo-γ-linolenic acid (DGLA; 20:3 n-6) and arachidonic acid (AOA; 20:4 n-6) in the n-6 family as well as eicosapentaenoic acid (EPA; 20:5 n-3) and docosahexaenoic acid (DHA; C22:6 n-3) in the n-3 family (39). Polyunsaturated fatty acids were further metabolized to specialized pro-resolving mediators (SPMs), and these SPMs have pro/anti-inflammation effects targeting inflammation resolution (40). For synthesis of n-3 an n-6 LC-PUFAs, eicosanoids and its related compounds, ALA and LIA compete each other for the same rate-limiting enzyme (FADS2) (41), and LA specifically binds to key enzymes of SPM synthesis, thus leads to imbalance in the inflammation resolution (40). It was also noted that different genetic background (FADS2, LOX) may affect the enzyme activity and impact downstream production of certain n-3 LC-PUFAs (42). Cod liver oil intake in first year baby (32), fatty acid status both in pregnant mothers (43) and infancy (44) were associated with the risk of t1dm. Increased supplementation of n-3 LC-PUFAs decreases mononuclear cell proliferation and interleukin 1β contents (45). High intake of fatty fish decreases the risk of diabetes conferred by GADA positivity (46), reduces the risk of LADA (47). Metabolomic profile study in patients with LADA did not find unique metabolite profile, LADA was therefore recognized metabolically an intermediate of t1dm and t2dm (48). LADA was more like t2dm, but overlapping t1dm, LADA progressed faster to insulin therapy (48). The metabolomic study did not centered on role of the LC-PUFAs, which are more closed related to immunity modulation. We therefore retrospectively retrieved data from our patients with diabetes who also had fatty acid profile analyzed during our clinic practice. Then we reclassified our patients into t1dm, LADA, and t2dm by age of onset, GADA positivity, and residual C-peptide. We found LADA patient had different fatty acid profile compared with t1dm and t2dm.

## Patients and Methods

### Data Collection and Diagnosis

Patients with diabetes admitted to our department during Jan 1, 2019 to Dec 31, 2022 were enlisted. Those were excluded when the patient was accompanied with critical illness, gestational diabetes, special types of diabetes, moderate to severe stage of renal failure and liver failure, malignancy, and disorders which might influence glycemia, i.e., Cushing syndrome, hyperthyroidism. Those who had missing data were also excluded from analysis. Physical measures, disease history and biochemical data of patients with fatty acids profile were retrospectively retrieved. Diagnosis of diabetes mellitus was according to criteria of World Health Organization. LADA was diagnosed as (a) positive GADA, (b) disease onset < 35 years old, and (c) fasting serum C-peptide (FCP) > 0.3 nmol/L (48). The patients were considered as type 1 diabetes if they were GADA positive and had a C-peptide < 0.3 nmol/L. The remaining patients was considered as having type 2 diabetes. 116 patients were GADA positive, 249 patients with type 2 diabetes were selected by Propensity Score Matching from 848 patients with type 2 diabetes. Blood samples of fasting after at least 8 hours post dinner, 1-hour and 2-hour post 100g bread intake were withdrawn to serum collecting tube and were sent to central laboratory within half an hour. Biochemistry analysis was undertaken in the Department of clinical laboratory of our hospital. C-peptide was determined by Immulite™ method (Siemens Healthcare, Deerfield, IL), GADA was measured by immunoluminescence sandwich method (Shenzhen New Industry Biomedical Engineering Co. LTD, Shenzhen, China). Patients with t1dm were younger (P=0.002), and have lower BMI (P< 0.001), lower level of 1-hour C-peptide (1hCP) (P<0.001), higher HbA1c level (P<0.001), were more insulin sensitive (as measured by C-peptide modified Matsuda index, ISI-CP) (P<0.001) and poor endogenous insulin secretion (as measured by mean C-peptide (0,1,2 hours)/glucose (0, 1, 2 hours), InSecrI-CP)(P<0.001). The age of onset was younger (P<0.001). FCP and 2-hour C-peptide (2hCP) were lowest in t1dm, highest in LADA (P<0.001). HDL-C was lowest in LADA, was highest in t12m (P<0.001). Urine albumin/Creatine was higher in LADA (P=0.009). No differences in fasting glucose (FBG), 1-hour glucose, and 2-hour glucose were observed among the three groups of diabetes (P>0.05). Frequency of antithyroid globulin autoantibodies (ATG) was 25.8% in t1dm, 10.6% in LADA, and 5.4% in t2dm respectively (P<0.001), and those of antithyroid peroxidase autoantibodies (ATPO) was 45.3% in t1dm, 21.3% in LADA, and 6.0% in type 2 diabetes respectively (P<0.001), there were no differences for diabetic retinopathy, nephropathy, coronary heart disease, and atherosclerosis among the three groups of diabetes (P>0.05). For hypoglycemic agents just before admission, the use of insulin was higher in patients with t1dm than t2dm and LADA, and similar between LADA and t2dm; The use of insulin secretagogues and metformin was less frequent in t1dm, and similar between LADA and t2dm (**Supplementary Table 1**). The study was approved by the local ethics committee and personal consent was exempt.

### Measurement of Fatty Acids

The methods in our analysis of fatty acid profile were described previously (48). In short, fasting blood samples at admission were drawn and stored at -80°C for the analysis of fatty acid lipid profiles. Reagents used: Water, acetonitrile and isopropyl alcohol, the agents were obtained from Fisher Scientific (Pittsburgh, PA, USA). Formic acid (>98%) and ammonium acetate (>99%) were obtained from Fluka (Buchs, Switzerland). Free fatty acids standards were obtained from Nu-Chek-Prep (Elysian, MN, USA). Samples were thawed at 4°C. Formic acid and ammonium acetate plus water or acetonitrile and isopropyl alcohol were used as the mobile phase. C19:0 was used as the internal standard. Liquid chromatography-tandem mass spectrometry analysis was carried out with Eksigent LC100 and AB SCIEX Triple TOF 5600 (AB SCIEX, Framingham, MA, USA). Eksigent LC100 was equipped with XBridge Peptide BEH C18 Column (Waters, Milford, MA, USA). PeakView1.2 (AB SCIEX) was used for qualitative analysis. MultiQuant2.1 (AB SCIEX) was used for quantitative analysis.

### Statistical Analysis

Variables with a skewed distribution were log-transformed prior to their statistical evaluation. Continuous variables are expressed as means ± standard deviations. Categorical variables are presented as frequencies (percentage). Differences between groups were assessed by ANOVA followed by adjustment for multiple comparisons using *post hoc* Tukey test, frequencies comparison of categorical variables were analyzed using Chi-square test. Principal factor analysis (PCA) was performed on mean centered and unit variance scaled data with Promax model of rotation. Analysis was carried out using SPSS version 26.0 (IBM, USA). R package software was used for heatmap drawing. *P*-values of <0.05 were considered statistically significant.

## Patients and Methods

### Factor Analysis of Fatty Acid Profile in Patients With Diabetes

We first preformed principal factor analysis to find out if there were specific combination of the fatty acid in patients with diabetes (**Supplementary Table 2**). Six components were found that explain 82.6% of total variance. By model of Promax rotation, we found that in component 1, the factor loading of myristoleic acid (MLA) (C14:1 n-5), palmitoleic acid (PLA) (C16:1 n-7), myristic acid (C14:0), eicosatrienoic acid (ETA) (C20:3 n-6), oleic acid (C18:1 n-9), eicosadienoic acid (EDA) (C20:2 n-6), decosatetraenoic acid (DTA) (C22:4 n-6), eicosenoic acid (ENA) (C20:1 n-9), ALA (C18:3 n-3), monounsaturated fatty acid (MUFA), decosapentaenoic acid (DPA) (C22:5 n-3) exceeded above 0.6, indicating a dietary intake of mixed lard oil, seafood and vegetable oil; In component 2, the factor loading of nervonic acid (NNA) (C24:1 n-9), behenic acid (C22:0), wood tar acid (WTA) (C24:0), arachidic acid (AIA) (C20:0), erucic acid (ECA) (C22:1 n-9), AOA (C20:4 n-6) exceeded 0.6, indicating dietary habit of vegetable oil; In component 3, the factor loading of eicosapentaenoic acid (EPA) (C20:5 n-3), decosahexaenoic acid (DHA) C22:6 n-3), n-3/n-6 ratio, and total n-3 PUFA exceeded 0.6, indicating a predominant seafood diet; In component 4, factor loading of total n-6 polyunsaturated fatty acid (PUFA), total PUFA, and total fatty acid (TFA) exceeded 0.6, indicating a high fat diet; In component 5, LIA (C18:2 n-6), palmitic acid (C16:0) exceeded 0.6, indicating a dietary intake of predominant vegetable oil; In component 6, factor loading of kwai acid (C19:0), lauric acid (C12:0) exceeded 0.6, again indicating a predominant dietary intake of vegetable oil again. The most interesting finding was the n-3 PUFA, especially those from seafood, such as EPA and DHA, the total n-3 PUFA level in serum and n-3/n-6 PUFA ratio clustered in one component.

### Comparison of Fatty Acid Profile in Different Groups of Diabetes

We then compared the differences of fatty acid profile in different groups of diabetes by ANOVA (**Supplementary Table 3**). Levels of PLA (C16:1 n-7) and ETA (C20:3 n-6) in LADA were higher than in t1dm and lower than in t2dm. Levels of lauric acid (C12:0), myristic acid (C14:0), ALA (C18:3 n-3), oleic acid (C18:1 n-9), EDA (C20:2 n-6), n-3/n-6 ratio, and total PUFA in LADA were higher than in t1em and like those in t2dm. Levels of kwai acid (C19:0), EPA (C20:5 n-3), AOA (C24:4 n-6), AIA (C20:0), DPA (C22:5 n-3), DTA (C22:4 n-6), ECA (C22:1 n-9), behenic acid (C22;0), NNA (C24:1 n-9), WTA (C24:0), Triene/tetraene ratio were higher in t2dm than in t1dm and LADA, they were not different between t1dm and LADA. Levels of ENA (C20:1 n-9) and Total n-3 PUFA in t2dm were higher than in t1dm, and not different between LADA and t2dm.

**Figure 1:**
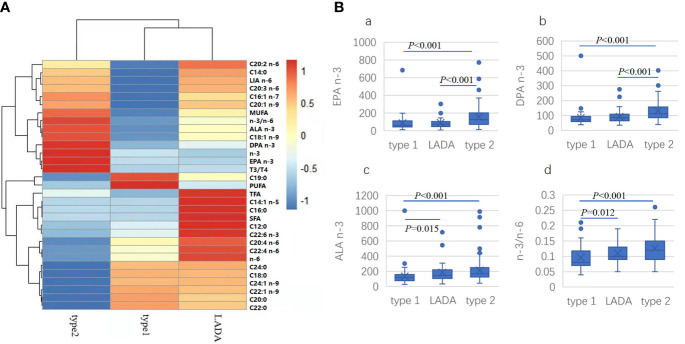
Heatmap drawn for comparison of the correlation of specific fatty acid with different types of diabetes **(A)**. LA: LIA (C18:2 n-6); MUFA, monounsaturated fatty acid; ALA (C18:3 n-3); DPA (C22:5 n-3); n-3, total n-3 PUFA; EPA, EPA (C20:5 n-3); T3/T4, triene/tetraene PUFA ratio; PUFA, polyunsaturated fatty acid; SFA: saturated fatty acid; n-6, total n-6 PUFA; Comparison of concentration of selected fatty acid indexes in different types of diabetes **(B)**. (a) EPA (C20:5 n-3), (b) DPA (C22: n-3). (c) ALA (C18:3 n-3), (d) n-3/n-6 PUFA ratio.

To find if there were unique picture of fatty acid profile can differentiate among the three groups of diabetes, heat map was drawn with R software (**Figure 1**). The three groups could be clearly separated. For t2dm, highest correlations were found in total n-3 PUFA, n-3/n-6 ratio, ALA (C18:3 n-3), DPA (C22:5 n-3), EPA (C20:5 n-3), triene/tetraene ratio, while the lowest correlations were found in stearic acid (C18:0), AIA (C20:0), behenic acid (C22:0), WTA (C24:0), ECA (C22:1 n-9) and NNA (C24:1 n-9). For t1dm, highest correlations were found in kwai acid and PUFA while lowest correlations were found in myristic acid (C14:0), PLA (C16:1 n-7), LA (C18:2 n-6), ENA (C20:1 n-9), EDA (C20:2 n-6) and ETA (C20:3 n-6). For LADA, highest correlations were found in lauric acid (C12:0), palmitic acid (C16:0), MLA (C14:1 n-5), EDA (C20:2 n-6). AOA (C20:4 n-6), DTA (C22:4 n-6), DHA (C22:6 n-3), total n-6 PUFA, TFA and SFA while lowest correlations were found in EPA (C20:5 n-3), total n-3 PUFA and triene/tetraene ratio. For n-3/n-6 ratio and DPA (C22:5 n-3), the highest correlation was found in t2dm, the lowest was in t1dm, intermediate in LADA. For EPA (C20:5 n-3), the highest correlation was found in t2dm, similar between t1dm and LADA. For total n-6 PUFA, the highest correlation was found in LADA, lowest in t2dm, intermediate in t1dm.

### Clustering of Fatty Acid Profile and its Distribution Among Different Groups of Diabetes

To study if different clusters of fatty acid profile distributed differently among different groups of diabetes mellitus, k-means clustering analysis found 6 clusters were achieved (**Supplementary Table 4**). Cluster 1, 4, and 6 contained too few samples and was excluded for further analysis. There were no differences in levels of kwai acid and lauric acid among cluster 2, 3, and 5, and all left fatty acid and indexes could differentiate among the three clusters of 2, 3, and 5.

ANOVA was conducted to compare if different clusters led to clinical characteristics (**Supplementary Table 4**). The BMI, waist circumference and systolic blood pressure (SBP) were lower in cluster 5 than those in cluster 3, and similar in cluster 2 and 3. Levels of HbA1c, total cholesterol (TChol), and LDL-C were lower in cluster 2 than in cluster 3 and 5, no differences were found between cluster 3 and 5. Insulin sensitivity was higher while residual β cell secretion was worse in cluster 5 than in cluster 2 and 3. Diastolic blood pressure (DBP) was lower in cluster 3 than that in cluster 2 and 5. Level of triglyceride was highest in cluster 3 among the three clusters, and that in cluster 5 was higher than in cluster 2.

Different types of diabetes distributed differently among the different clusters of fatty acid profile (**Supplementary Table 6**). 129 patients (50%) distributed in cluster 2, 38 (14.7%) in cluster 3, 85 (32.9%) in cluster 5. In cluster 3, 38 patients were solely t2dm. Cluster 2 contained 26 patients with t1dm (28.3%), 13 LADA (28.3%), and 90 t2dm (60.4%), Cluster 5 contained 36 t1dm (57.1%), 30 LADA (65.2%), and 19 t2dm (12.8%).

### Correlation of Fatty Acid With Autoimmunity and β Cell Function

Spearman correlation analysis was conducted to find out the association of fatty acid profile with β cell autoimmunity, thyroid autoimmunity (ATG and ATPO), insulin sensitivity and endogenous insulin secretion indexes (**Supplementary Table 7**).

Kwai acid (C19:0), myristoleic acid (MLA) (C14:1 n-5), AOA (20:4 n-6), AIA (C20:0), ECA (C22:1 n-9), behenic acid (C22:0), NNA (C24:1 n-9), WTA (C24:0) are positively, and myristic acid (C14:0), PLA (C16:1 n-7), ALA (18:3 n-3), oleic acid (18:1 n-9), EPA (C20:5 n-3), ETA (C20:3 n-6), EDA (C20:2 n-6), ENA (C20:1 n-9), DPA (C22:5 n-3), n-3 PUFA, MUFA, n-3/n-6 ratio, Triene/tetraene ratio negatively correlated with GADA. Similar findings were also observed for ATG and/or ATPO.

Myristic acid (C14:0), PLA (C16:1 n-7), palmitic acid (C16:0), LA (18:2 n-6), oleic acid (18:1 n-9), AOA (C20:4 n-6), DTA (C22:4 n-6), behenic acid (C22:0), WTA (C24:0), n-6 PUFA, PUFA, SFA,TFA were positively, and ETA (C20:3 n-6) and Triene/tetraene ratio were negatively correlated with glycemic control (HbA1c or FBG or 2hBG), especially palmitic acid (C16:0) and DTA (C22:4 n-6) were correlated positively with all the three glycemic control indexes. n-3 PUFA was not associated while n-6 PUFA was associated positively with glycemic control (HbA1c or FBG or 2hBG).

Kwai acid (C19:0), AIA (C20:0), ECA (C22:1 n-9), behenic acid (C22:0), nervonic acid (C24:1), WTA (C24:0) were negatively, and MLA (C14:1 n-5), myristic acid (C14:0), PLA (C16:1 n-7), palmitic acid (C16:0), ALA (C18:3 n-3), oleic acid (18:1 n-9), EPA (C20:5 n-3), ETA (C20:3 n-6), EDA (C20:2 n-6), ENA (C20:1 n-9), DPA (C22:5 n-3), ECA (C22:1 n-9), Triene/tetraene ratio, n-3/n-6 ratio, n-3 PUFA, MUFA, SFA and TFA were positively correlated with insulin sensitivity. Myristic acid (C14:0), PLA (C16:1 n-7), ALA (C18:3 n-3), Oleic acid (18:1 n-9), EPA (C20:5 n-3), ETA (C20:3 n-6), DPA (C22:5 n-3), n-3/n-6 ratio, triene/tetraene ratio and MUFA were positively, and kwai acid (C19:0), AOA (20:4 n-6), AIA (C20:0), ECA (C22:1 n-9), behenic acid (C22:0), NNA (C24:1 n-9), WTA (C24:0) were negatively correlated with β cell function.

As for clinical implications, all the association were low. A correlation higher than 0.5 might be of clinically significance. Arachidic acid (C20:0) (r=0.602), ECA (C22:1 n-9) (r=0.557), behenic acid (C22:0) (r=0.633), NNA (C24:1 n-9) (r=0.636), WTA (C24:0) (r=0.632) were positively, and triene/tetraene ratio (r=-656) was negatively associated with the presence of GADA.

## Discussion

In our study we found that patients with diabetes can be grouped into six principal factors, indicating a predominantly mixed dietary intake, predominantly vegetable oil users, seafood users (49). The overall picture of fatty acid profile in t2dm compared to t1dm and LADA was associated with higher concentration or high correlation of n-3/n-6 ratio, triene/tetraene ratio and decreased saturated fatty acid. The overall picture in t1dm was associated with increased total PUFA and kwai acid, and decreased n-3/n-6 ratio. The overall picture in LADA was associated with increased TFA, SFA, and decreased n-3 PUFA, more intermediate between t1dm and t2dm. Although 6 clusters were achieved, but only three contained enough samples could be used for analysis. Cluster 5 like mostly insulin deficient type of diabetes accounted nearly 60% of patients with t1dm and more than 60% of LADA, only 12.8% of t2dm, cluster 2 and 3 like more t2dm with cluster 3 contained only patients, accounted 25.5% of patients with t2dm, and cluster 2 contained more than 60% of patients with t2dm, and only 28% of patients with t1dm and LADA respectively. Saturated fatty acid and AOA (C20:4 n-6) was positively, while MUFA, n-3 PUFA, especially n-3/n-6 ratio and triene/tetraene ratio were negatively associated negatively with GADA and thyroid autoimmunity. Similar findings were found for residual β cell function. Saturated fatty acid was associated with insulin resistance, while n-3 PUFA, n-6 PUFA, MUFA, especially n-3/n-6 ratio and triene/tetraene ratio were positively associated with insulin sensitivity.

With the easy availability and promotion, the modern diet is high in saturated fat and n-6 (ω-6) polyunsaturated fatty acids (PUFAs) (mainly from vegetable oil) and low in long chain n-3 (ω-3) PUFAs (mainly from seafood). In addition, more than half of the calories consumed in the modern diet come from highly processed foods (50). The current ratio of n-6 to n-3 PUFAs intake has significantly increased from 1:1 to 10:1 today (51). This may be due to the use of vegetable oils and processed foods (52, 53). As with the increase intake high fat diet (milk, fatty meat, and vegetable oil), diabetes prevalence increased as manifested from 0.67% to 12.6% in China (53). The promotion of increased n-3 PUFA on health is being consistent approached while the promotion of increased intake n-6 PUFA is being debated (54, 55). Current view on supplementation of PUFA to improve health is to increase the ratio of n-3/n6 PUFA rather than solely increase PUFA. More recently dietary PUFA and genetic interaction might play an important role in modulation of production of PUFA biological metabolites (54, 55). Therefore, the physiological roles of PUFA are very complicated.

Although heatmap analysis showed a clear distinct picture among the three groups of diabetes, but like the study of Al-Majdoub et al. (21), there was no unique combination of fatty acid indexes. Distribution of groups of diabetes in clusters of fatty acid profile also could not separate the three groups of diabetes completely, indicating again LADA is mixture of t1dm and t2dm.

Autoimmunity is another feature of and prerequisite of diagnosing LADA (8). It is interesting to study the reason why pancreas β cell failure progresses slower than classical t1dm. Many hypothetic views were proposed (56), such HLA genotypes, risk genotype of t2dm (57), distorted innate inflammation (30), etc. PUFA, especially n-3, n-6 PUFA are important precursors of specialized pro-resolving mediators (SPMs) (41). Apart from intake of ALA (C18:3 n-6) and AIA (C18:2 n-6) rich plant oils, n-3, n-6 PUFA should be taken from fatty fish (seafood), therefore, n-3, n-6 PUFA are named essential fatty acid (37, 38). High fatty fish intake not only associated decreased GADA development (46), but also decreased the incidence of LADA. Autoimmunity should be fine-tuned between proinflammatory and anti-inflammatory states (30). SPMs from n-3 and n-6 PUFA undergoing through different pathway results different SPMs specialized in modulation proinflammatory or anti-inflammatory actions. Although it is recognized n-3 PUFA mainly lead to anti-inflammatory action, n-6 PUFA can also modulate the process of inflammation resolution (40). Apart from competing between n-3 and n-6 PUFAs for elongation and desaturation, increased intake of LIA (C18:2 n-6) can compete key enzymes for SPMs biosynthesis, thus disturb the process of inflammation fine-tuning (40). Our study also found ALA (C18:3 n-3) and n-3 PUFAs, such as EPA (20:5 n-3), DPA (22:5 n-3), some n-6 PUFAs such as ETA (C20:3 n-6), EDA (C20:2 n-6) were associated negatively with GADA, some n-6, such as AOA (C18:2 n-6), DTA (C22:4 n-6) were positively associated GADA while the n-3/n-6 ratio and triene/tetraene were negatively associated with GADA, indicating not only in higher proportion of n-3, but also the ratio of n-3/n-6 ratio and triene/tetraene ratio are also important. Moreover, we also found behenic acid (C22:0), NNA (C24:1 n-9), WTA (C24:0), which might come from contamination in vegetable oil (58), were associated highly with GADA positivity. Taking together with similarly high association of AIA (C18:2 n-6) which also comes from plant oil, it would be supposed that high intake of vegetable oil rich in AIA (C18:2 n-6), either through competing with ALA (C18:3 n-3) or inhibiting the enzymes of SPMs biosynthesis, might increase the development autoimmunity. It is interesting to mention here the study of Pflueger et al. (59) and Frederiksen et al. (60). Pflueger found islet autoimmunity is associated with differences in lipid and amino acid concentrations, and interestingly in children with early onset of islet autoimmunity have markedly lower concentration of methionine (59). Frederiksen found erythrocyte membrane n-3 fatty acid level was not inversely associated with islet autoimmunity only after age 4.25 years old (60). These studies might indicate that early onset of autoimmunity might result from more genetic susceptibility related to protein metabolism while late onset islet autoimmunity comes from subtly insidious abnormalit(ies), such as in dietary fatty acid metabolism, especially in SPMs production.

Chronic inflammation is associated insulin resistance (61). High fat diet increases obesity and leads to low level of chronic inflammation (62). The increase intake n-6 PUFA parallel the prevalence of adiposity. Increase n-3 PUFA improves insulin sensitivity (63) while increased intake n-6 decreases insulin sensitivity (64). In Our study we found ALA (C18:3 n-3) and n-3 PUFAs, such as EPA (C20:5 n-3), DPA (C22:5 n-3) and some n-6 PUFAs, such as ETA (C20:4 n-6), EDA (C20:2 n-6) and DTA (C22:4 n-6) were associated with increased insulin sensitivity, while higher n-3/n-6 ratio and triene/tetraene ratio had similar associations. For residual β cell function, similar associations were found. Although n-3 and n-6 could improve insulin sensitivity and β cell function, the influence on glycemic control are completely different. We found in our study although palmitic acid (C16:0) and DTA (C22:4 n-6) were associated increased insulin sensitivity, but associated increase HbA1c, FBG and/or 2hBG. These two fatty acids had no effect on improvement of β cell function, indicating the β cell function as a dominant factor in glycemic control.

The short comings of our study were (a) the population selected was from routine clinical practice, (b) the hypoglycemic treatment modality, especially the time of insulin therapy after diagnosis, was not recorded, (c) only GADA was used in selection of LADA. As the start of insulin therapy for non-insulin dependent patients with diabetes was usually empirical, we use the residual fasting C-peptide > 0.3 nmol/L as alternative as Al-Majdoub et al. (21). Because we do not check autoantibodies other than GADA and GADA was the most frequent autoantibodies and last longer than other autoantibodies, therefore, use GADA as the index to diagnose LADA is also acceptable alternative. (d) lastly, insulin therapy, use of secretagogues agents and metformin were found different among the three group. The different use of therapeutic modalities were intrinsic flaws. In study of Hodge et al. (65), they found smoking influence the level of phospholipid DHA, but fasting or not, hypoglycemic treatment, statin use did not affect the measurement of fatty acid. We therefore did not correct the use of antidiabetic agents or statins in analysis.

In summary, we studied fatty acid profile in patients with t1dm, t2dm and LADA with aim to delineate if there were any unique feature that could be used as differentiation marker for intriguing subtype of autoimmune diabetes. To our knowledge, our study might be the first study to look specifically the association of fatty acid to LADA. Increased intake of n-3, some n-6, especially products that contain higher n-3/n-6 ratio and triene/tetraene ratio might be beneficial to protect from development of diabetes associated autoantibodies. Increased intake of impurified vegetable oil that has higher n-6 PUFA might counteract the beneficial health promoting of n-3 PUFA. Although our heatmap picture looked clearly different among the three groups of diabetes, the fatty acid profile of patients with LADA was still at the intermediate in between t1dm and t2dm, we could not find unique feature that can be used to subtype LADA from t1dm or t2dm. More well-designed clinical trials are still needed in delineating the role of PUFAs to prevent or slow dawn the speed of β cell deterioration in patients with LADA.

## Data Availability Statement

The raw data supporting the conclusions of this article will be made available by the authors, without undue reservation.

## Ethics Statement

The studies involving human participants were reviewed and approved by Ethics committee of Second Hospital of Dalian Medical Univeristy, No. 467, Zhongshan Rd, Shahekou District, Dalian 116027, China. The patients/participants provided their written informed consent to participate in this study.

## Author Contributions

HT, SW, YD, YX collected data and conducted parts of the data analysis, NL and BS designed the study and drafted the manuscript, LZ, XZ, PZ took care of the patients. All the authors have read and approved the submission of the final version of the manuscript which meets at least one of the following criteria recommended by the ICMJE* (http://www.icmje.org/recommendations/): 1. substantial contributions to conception and design, acquisition of data or analysis and interpretation of data; 2. drafting the article or revising it critically for important intellectual content.

## Conflict of Interest

The authors declare that the research was conducted in the absence of any commercial or financial relationships that could be construed as a potential conflict of interest.

## Publisher’s Note

All claims expressed in this article are solely those of the authors and do not necessarily represent those of their affiliated organizations, or those of the publisher, the editors and the reviewers. Any product that may be evaluated in this article, or claim that may be made by its manufacturer, is not guaranteed or endorsed by the publisher.
