# Supplementary material for: Fatty Acid Profiles and Their Association With Autoimmunity, Insulin Sensitivity and β Cell Function in Latent Autoimmune Diabetes in Adults
Source: Front Endocrinol (Lausanne). 2022 Jun 29;13:916981. doi: 10.3389/fendo.2022.916981 (PMC9276921; doi:10.3389/fendo.2022.916981)
Supplement: Supplementary file 1 [file DataSheet_1.zip › Supplementary Table 1.docx]

Supplementary Table 1 Clinical measures and biochemical data

| Indexes | Type 1 | LADA | Type 2 | F/χ2 | *P* |
| --- | --- | --- | --- | --- | --- |
| n (male%) | 64(45.3) | 47(48.9) | 149(49.0) | 0.26 | 0.878 |
| Age (yr) | 52.66±16.85 | 58.96±12.07 | 59.34±12.28^a^ | 6.277 | **0.002** |
| Age at dx (yr) | 43.87±13.93 | 50.75±9.52^a^ | 49.35±10.40^a^ | 6.754 | **0.001** |
| DD (yr) | 8.81±8.60 | 8.36±7.16 | 9.99±7.57ab | 8.6 | **0.001** |
| BMI (kg/m2) | 22.17±3.53 | 26.54±3.35^a^ | 25.68±3.64^a^ | 26.219 | **0.001** |
| Waist (cm) | 82.63±11.72 | 93.91±14.30^a^ | 92.41±9.92^a^ | 19.16 | **0.001** |
| SBP (mmHg) | 133.78±21.45 | 143.38±17.77 | 148.19±22.30^a^ | 10.218 | **0.001** |
| DBP (mmHg) | 80.11±10.95 | 81.13±11.13 | 82.04±11.52 | 0.669 | 0.513 |
| Crea (μmol/L) | 57.06±14.10 | 64.15±20.20 | 62.13±17.01 | 2.793 | 0.063 |
| eGFR(ml) | 89.32±4.12 | 87.15±8.12 | 88.19±6.70 | 1.528 | 0.229 |
| UA (μmol/L) | 283.00±97.81 | 347.88±90.19^a^ | 320.82±89.89^a^ | 7.028 | **0.001** |
| Alb/Cre (μg/g) | 94.65±232.50 | 192.70±500.03^a^ | 58.79±121.30^b^ | 4.794 | **0.009** |
| A1C (%) | 9.64±2.49 | 8.51±2.28^a^ | 8.34±1.88^a^ | 8.6 | **0.001** |
| FBG (mmol/L) | 9.06±4.56 | 8.97±3.34 | 8.98±3.05 | 0.013 | 0.987 |
| Fins (μIU/L) | 15.17±15.32 | 29.43±86.14 | 14.13±15.06 | 2.261 | 0.106 |
| FCP (nmol/L) | 0.127±0.092 | 0.545±0.235^a^ | 0.445±0.219^ab^ | 126.66 | **0.001** |
| 1hBG (mmol/L) | 13.50±4.48 | 14.26±4.29 | 13.36±3.96 | 0.702 | 0.496 |
| 1hIns (μIU/L) | 44.51±35.19 | 47.56±34.05 | 44.28±32.20 | 1.827 | 0.163 |
| 1hCP (ng/ml) | 0.263±0.329 | 0.997±0.511^a^ | 0.880±0.528^a^ | 106.525 | **0.001** |
| 2hBG (mmol/L) | 15.45±6.39 | 15.17±5.04 | 14.00±4.93 | 2.02 | 0.135 |
| 2hIns (μIU/L) | 36.70±24.61 | 52.46±35.80^a^ | 48.49±36.56^a^ | 6.673 | **0.001** |
| 2hCP (ng/ml) | 0.350±0.385 | 1.412±0.712^a^ | 1.18±0.700^ab^ | 99.538 | **0.001** |
| Chol (mmol/L) | 5.19±1.37 | 4.96±1.45 | 5.05±1.23 | 0.458 | 0.633 |
| Trig (mmol/L) | 1.53±2.05 | 2.17±1.68^a^ | 2.03±2.94^a^ | 9.179 | **0.001** |
| HDL-C (mmol/L) | 1.40±0.36 | 1.12±0.26^a^ | 1.23±0.30^ab^ | 11.706 | **0.001** |
| LDL-C (mmol/L) | 2.82±0.90 | 2.81±1.11 | 2.66±0.71 | 1.118 | 0.329L |
| IglSI-CP | 3.51±0.64 | 2.62±0.18^a^ | 2.73±0.22^a^ | 117.368 | **0.001** |
| lgInsSecr-CP | -1.53±0.67 | -0.66±0.25^a^ | -0.73±0.29^a^ | 96.94 | **0.001** |
| ATG (%) | 16(25.8) | 5(10.6) | 8(5.4) | 18.35 | **0.001** |
| ATPO (%) | 27(45.3) | 10(21.3) | 9(6.0) | 42.509 | **0.001** |
| diabetic retinopathy (%) | 7(36.8) | 2(12.5) | 28(18.8) | 4.049 | 0.132 |
| diabetic nephropathy (%) | 9(40.0) | 6(37.5) | 37(24.8) | 2.897 | 0.235 |
| CHD (%) | 1(5.3) | 1(6.3) | 11(7.4) | 0.133 | 0.936 |
| Atherosclerosis (%) | 15(78.9) | 15(93.6) | 101(67.8) | 5.371 | 0.068 |
| Hypoglycemic agents |  |  |  |  | 0.898 |
| Yes/no/NA (n) | 54/9/1 | 38/7/2 | 136/21/1 | 2.225 | 0.898 |
| Insulin (%) | 73.0 | 26.7 | 39.5 | 31.426 | **0.001** |
| Insulin secretagogues (%) | 7.9 | 28.9 | 21.1 | 9.135 | **0.028** |
| Metformin (%) | 19.0 | 46.7 | 49.7 | 21.066 | **0.001** |
| Alpha-glucosidase inhibitors (%) | 27.0 | 26.7 | 32.7 | 3.168 | 0.367 |
| Thiazolidinediones (%) | 6.3 | 6.7 | 2.7 | 2.140 | 0.448 |
| Sodium-glucose cotransporter 2 inhibitors (%) | 0 | 8.9 | 4.1 | 5.718 | 0.126 |
| dipeptidyl peptidase 4 inibitors | 1.6 | 6.7 | 7.5 | 3.687 | 0.297 |
| GLP-1 RAs | 0 | 0 | 2.1 | 2.384 | 0.497 |

*DD: duration of diabetes; ATPO: antithyroid peroxidase autoantibodies, ATG: 31.426antithyroid globulin autoantibodies; CHD: coronary heart disease; lgISI-CP: log transformed Matsuda index modified by C-peptide; lgInsSecr-CP: Log transformed insulin secretion index computed with sum of 0, 1h, 2h CP over sum of 0, 1h, 2h glucose. a: compared with type 1 diabetes, b compared with type 2 diabetes. LADA: latent autoimmune diabetes in adults.

cid, LIA: Linoleic Acid.
