# Supplementary material for: Fatty Acid Profiles and Their Association With Autoimmunity, Insulin Sensitivity and β Cell Function in Latent Autoimmune Diabetes in Adults
Source: Front Endocrinol (Lausanne). 2022 Jun 29;13:916981. doi: 10.3389/fendo.2022.916981 (PMC9276921; doi:10.3389/fendo.2022.916981)
Supplement: Supplementary file 1 [file DataSheet_1.zip › Supplementary Table 2.docx]

Supplementary Table 2. Model array from PCA and factor analysis by model of Promax rotation

|  | factors | | | | | |
| --- | --- | --- | --- | --- | --- | --- |
|  | 1 | 2 | 3 | 4 | 5 | 6 |
| Myristoleic acid (C14:1 n-5) | **0.974** | 0.290 | -0.042 | -0.138 | -0.321 | 0.155 |
| Palmitoleic acid (C16:1 n-7) | **0.966** | -0.136 | 0.060 | -0.146 | 0.024 | -0.003 |
| Myristic acid (C14:0) | **0.860** | -0.030 | 0.052 | 0.018 | -0.065 | 0.156 |
| ETA (C20:3 n-6) | **0.830** | -0.325 | 0.004 | -0.329 | 0.133 | -0.244 |
| Oleic acid (C18:1 n-9) | **0.802** | -0.103 | -0.065 | 0.039 | 0.261 | -0.017 |
| EDA (C20:2 n-6) | **0.765** | -0.035 | -0.072 | 0.249 | 0.090 | 0.018 |
| DTA (C22:4 n-6) | **0.753** | 0.405 | -0.180 | 0.110 | 0.005 | -0.208 |
| ESA (C20:1 n-9) | **0.731** | 0.021 | 0.028 | 0.284 | -0.063 | 0.079 |
| ALA n-3 (C18:3 n-3) | **0.709** | -0.071 | 0.117 | 0.192 | 0.039 | 0.001 |
| MUFA | **0.645** | -0.049 | -0.002 | 0.492 | -0.053 | -0.064 |
| DPA (C22:5 n-3) | **0.636** | -0.326 | 0.266 | 0.196 | 0.041 | -0.095 |
| SFA | 0.448 | 0.123 | -0.024 | 0.441 | 0.189 | 0.088 |
| Stearic acid (C18:0) | 0.411 | 0.224 | 0.091 | 0.091 | 0.320 | 0.160 |
| Nervonic acid (C24:1 n-9) | -0.074 | **0.971** | 0.015 | -0.006 | -0.037 | -0.040 |
| Behenic acid (C22:0) | -0.007 | **0.953** | -0.085 | -0.052 | 0.042 | 0.041 |
| WTA (C24:0) | -0.002 | **0.947** | -0.070 | -0.018 | 0.032 | 0.020 |
| Triene/Tetraene | 0.459 | **-0.889** | -0.070 | -0.024 | 0.059 | 0.078 |
| AIA (C20:0) | 0.192 | **0.863** | -0.095 | 0.004 | 0.020 | 0.123 |
| ECA (C22:1 n-9) | 0.316 | **0.691** | 0.013 | 0.203 | -0.326 | 0.015 |
| AOA (C20:4 n-6) | 0.071 | **0.616** | 0.229 | 0.027 | 0.246 | -0.281 |
| n-3/n-6 | 0.149 | -0.003 | **1.044** | -0.356 | -0.291 | 0.039 |
| EPA (C20:5 n-3) | -0.090 | -0.223 | **0.932** | 0.218 | -0.094 | 0.112 |
| DHA C22:6 n-3) | 0.016 | 0.257 | **0.703** | 0.140 | 0.167 | -0.021 |
| Total n-3 | 0.245 | 0.032 | **0.610** | 0.392 | -0.027 | -0.006 |
| Total n-6 | 0.158 | 0.021 | -0.059 | **0.804** | 0.223 | -0.042 |
| PUFA | 0.212 | 0.006 | 0.034 | **0.749** | 0.192 | -0.047 |
| TFA | 0.326 | 0.033 | -0.035 | **0.588** | -0.013 | -0.073 |
| LIA (C18:2 n-6) | 0.033 | -0.172 | -0.256 | 0.353 | **0.872** | 0.139 |
| Palmitic acid (C16:0) | 0.443 | 0.197 | -0.039 | -0.081 | **0.572** | 0.141 |
| Kwai acid (C19:0) | -0.107 | 0.125 | 0.209 | -0.328 | 0.365 | **0.732** |
| Lauric acid (C12:0) | 0.231 | -0.119 | -0.023 | 0.149 | 0.000 | **0.695** |

Note: ETA: Eicostrienoic acid, EDA: Eicosadienoic acid, ESA: Eicosenoic acid, ALA: Linolenic acid, MUFA: monounsaturated fatty acid, DPA: docosapentaenoic Acid, SFA: total saturated fatty acid, WTA: wood tar acid, AIA: Arachidic acid, ECA: Erucic Acid, AOA: Arachidonic Acid, EPA: Eicosapentaenoic Acid, DHA: Docosahexaenoic Acid, PUFA: polyunsaturated fatty acid, TFA: total fatty acid, LIA: Linoleic Acid.
