# Supplementary material for: Fatty Acid Profiles and Their Association With Autoimmunity, Insulin Sensitivity and β Cell Function in Latent Autoimmune Diabetes in Adults
Source: Front Endocrinol (Lausanne). 2022 Jun 29;13:916981. doi: 10.3389/fendo.2022.916981 (PMC9276921; doi:10.3389/fendo.2022.916981)
Supplement: Supplementary file 1 [file DataSheet_1.zip › Supplementary Table 3.docx]

Supplementary Table 3 Comparison of fatty acid profile among different types of diabetes mellitus

| Fatty acid | Type 1 | LADA | Type 2 | *F* | *P* |
| --- | --- | --- | --- | --- | --- |
| Kwai acid (C19:0) | 7.46±4.10 | 7.77±4.54 | 6.53±6.38^ab^ | 5.332 | **0.050** |
| Lauric acid (C12:0) | 14.16±11.30 | 19.79±15.33^a^ | 19.46±27.02^a^ | 4.221 | **0.016** |
| Myristoleic acid (C14:1 n-5) | 5.48±5.46 | 7.19±6.56^a^ | 5.89±6.35^b^ | 4.461 | **0.012** |
| Myristic acid (C14:0) | 55.78±73.16 | 93.19±77.25^a^ | 96.26±87.24^a^ | 13.402 | **0.004** |
| Palmitoleic acid (C16:1 n-7) | 136.16±95.97 | 193.28±126.97^a^ | 246.40±167.45^ab^ | 22.667 | **0.001** |
| Palmitic acid (C16:0) | 3599.28±1109.40 | 3959.79±1152.03 | 3617.15±1193.75 | 1.72 | 0.181 |
| Linolenic acid (C18:3 n-3) | 135.86±124.52 | 186.13±147.18^a^ | 212.50±144.58^a^ | 15.793 | **0.001** |
| Linoleic Acid (C18:2 n-6) | 4506.32±982.53 | 4578.81±1150.44 | 4643.40±979.63 | 0.467 | 0.627 |
| Oleic acid (C18:1 n-9) | 1894.22±930.73 | 2428.19±1206.67^a^ | 2621.22±1232.83^a^ | 12.566 | **0.001** |
| Stearic acid (18:0) | 1158.72±398.54 | 1218.79±452.40 | 1124.56±417.50 | 1.302 | 0.302 |
| Eicosapentaenoic Acid (C20:5 n-3) | 86.72±90.27 | 90.06±66.57 | 157.83±119.31^ab^ | 22.806 | **0.001** |
| Arachidonic Acid (C24:4 n-6) | 1321.19±417.48 | 1396.25±479.21 | 1049.77±429.24^ab^ | 17.5 | **0.001** |
| Eicostrienoic acid (C20:3 n-6) | 96.72±49.13 | 157.62±74.49^a^ | 190.97±96.03^ab^ | 44.742 | **0.001** |
| Eicosadienoic acid (C20:2 n-6) | 28.80±18.91 | 37.73±18.69^a^ | 37.29±19.42^a^ | 11.197 | **0.009** |
| Eicosenoic acid (C20:1 n-9) | 16.07±13.35 | 18.52±14.82 | 19.43±12.57^a^ | 4.539 | **0.012** |
| Arachidic acid (C20:0) | 37.34±21.96 | 35.75±19.88 | 13.13±16.54^ab^ | 70.908 | **0.001** |
| Docosahexaenoic Acid (C22:6 n-3) | 370.14±169.79 | 425.15±219.68 | 384.90±182.54 | 1.241 | 0.291 |
| Docosapentaenoic Acid (C22:5 n-3) | 84.84±59.31 | 91.49±44.49 | 131.41±67.14^ab^ | 26.233 | **0.001** |
| Docosatetraenoic acid (C22:4 n-6) | 21.61±12.46 | 26.38±14.71 | 18.45±11.28^ab^ | 12.206 | **0.001** |
| Erucic Acid (C22:1 n-9) | 4.86±3.78 | 5.22±4.77 | 2.12±3.53^ab^ | 49.832 | **0.001** |
| behenic acid (C22;0) | 54.50±29.28 | 53.76±26.69 | 12.95±23.19^ab^ | 116.09 | **0.001** |
| Nervonic acid (C24:1 n-9) | 75.52±36.87 | 79.66±31.60 | 17.20±34.87^ab^ | 144.39 | **0.001** |
| Wood tar acid (C24:0) | 50.35±26.52 | 50.47±25.75 | 11.93±21.75^ab^ | 105.93 | **0.001** |
| ω3/ω6 | 0.096±0.036 | 0.134±0.171^a^ | 0.126±0.044^a^ | 12.112 | **0.031** |
| Triene/tetraene | 0.051±0.128 | 0.043±0.109 | 0.364±0.222^ab^ | 92.19 | **0.001** |
| Total saturated fatty acid | 5375.64±3467.33 | 5542.92±1847.24 | 5145.12±2112.95 | 0.109 | 0.896 |
| Total monounsaturated fatty acid | 2387.66±2781.02 | 2920.85±1529.32^a^ | 2953.28±1529.32^a^ | 8.725 | **0.001** |
| Total poly unsaturated fatty acid | 7330.77±4870.90 | 7406.89±2946.36 | 7164.44±2387.16 | 0.125 | 0.882 |
| Total ω3 | 612.20±520.18 | 718.32±441.20 | 754.95±372.35^a^ | 6.757 | **0.001** |
| Total ω6 | 6471.35±4246.28 | 6373.38±2456.43 | 6029.77±2028.43 | 0.664 | 0.516 |
| Total fatty acid | 15062.98±10935.48 | 18040.75±15636.08 | 15201.20±5640.47 | 1.74 | 0.178 |
