# Supplementary material for: Fatty Acid Profiles and Their Association With Autoimmunity, Insulin Sensitivity and β Cell Function in Latent Autoimmune Diabetes in Adults
Source: Front Endocrinol (Lausanne). 2022 Jun 29;13:916981. doi: 10.3389/fendo.2022.916981 (PMC9276921; doi:10.3389/fendo.2022.916981)
Supplement: Supplementary file 1 [file DataSheet_1.zip › Supplementary Table 4.docx]

Supplementary Table 4. Clustering of fatty acid profile

|  |  | Numbers of cluster | | | | |  |  |
| --- | --- | --- | --- | --- | --- | --- | --- | --- |
|  | 1 | 2 | 3 | 4 | 5 | 6 |  |  |
| n | 1 | 130 | 38 | 1 | 89 | 4 | F | P |
| kwai acid (C19:0) | 4 | 6.29±6.31 | 7.33±5.28 | 18 | 7.43±4.29^a^ | 9.75±5.25 | 1.6 | 0.16 |
| lauric acid (C12:0) | 80 | 17.20±23.22 | 21.80±33.10 | 4 | 17.88±12.38 | 29.00±9.20 | 2.118 | 0.064 |
| myristoleic acid (C14:1 n-5) | 39 | 3.85±3.17 | 10.21±9.55^a^ | 7 | 6.42±3.52^a^ | 25.00±13.09 | 31.265 | **0.001** |
| Myristic acid (C14:0) | 600 | 54.90±41.74 | 183.08±112.04^a^ | 145 | 75.33±41.92^ab^ | 297.25±136.66 | 53.703 | **0.001** |
| palmitoleic acid (C16:1 n-7) | 661 | 151.69±66.45 | 449.97±195.32^a^ | 155 | 176.15±79.45^b^ | 556.26±199.90 | 66.129 | **0.001** |
| palmitic acid (C16:0) | 6000 | 2900.29±671.62 | 4892.84±990.23^a^ | 3958 | 4226.30±958.06^ab^ | 6000±0.00 | 55.254 | **0.001** |
| lenolenic acid (C18:3 n-3) | 1000 | 130.38±50.69 | 361.37±154.21^a^ | 172 | 167.02±79.58^ab^ | 725.75±227.21 | 91.626 | **0.001** |
| lenoleic acid (C18:2 n-6) | 6000 | 4051.95±858.32 | 5547.45±711.86^a^ | 3329 | 4954.10±805.42^ab^ | 6000.00±0.00 | 28.793 | **0.001** |
| oleic acid (C18:1 n-9) | 6000 | 1846.33±595.05 | 4089.82±1154.48^a^ | 1397 | 2347.80±886.76^ab^ | 5791.75±241.17 | 65.211 | **0.001** |
| Stearic acid (C18:0) | 3000 | 919.19±226.33 | 1465.71±457.99^a^ | 925 | 1292.60±322.90^a^ | 1153.35±418.34 | 44.285 | **0.001** |
| Eicosapentaenoic Acid (C20:5 n-3) | 685 | 110.74±88.02 | 223.71±157.50^a^ | 81 | 102.75±69.09^b^ | 215.75±85.07 | 17.143 | **0.001** |
| Arachidonic Acid (C 20:4 n-6) | 2500 | 893.67±286.65 | 1257.84±367.64^a^ | 809 | 1522.18±355.21^ab^ | 2334.75±330.50 | 54.214 | **0.001** |
| Eicosatrienoic acid (C20:3 n-6) | 155 | 141.01±69.97 | 295.32±107.27^a^ | 98 | 133.79±56.11^b^ | 216.75±83.02 | 31.091 | **0.001** |
| Eicosadienoic acid (C20:2 n-6) | 147.5 | 26.96±8.52 | 54.96±17.67^a^ | 27.1 | 33.58±10.34^ab^ | 116.93±36.19 | 94.977 | **0.001** |
| eicosenoic acid (C20:1 n-9) | 100 | 13.52±4.73 | 30.85±16.42^a^ | 12.9 | 16.96±6.28^ab^ | 72.90±19.62 | 81.659 | **0.001** |
| arachidic acid (C20:0) | 100 | 11.59±11.27 | 11.26±10.43 | 43 | 41.17±16.84^ab^ | 100.00±0.00 | 92.423 | **0.001** |
| decosahexaenoic acid (C22:6 n-3) | 1000 | 296.59±99.45 | 497.21±215.58^a^ | 204 | 447.97±170.79^a^ | 388.82±186.11 | 31.679 | **0.001** |
| decosapentaenoic acid (C22:5 n-3) | 500 | 91.56±35.08 | 214.71±68.55^a^ | 68 | 90.76±23.60^b^ | 244.50±22.88 | 96.296 | **0.001** |
| decosatetraenoic acid (C22:4 n-6) | 100 | 13.76±4.90 | 27.74±10.73^a^ | 24 | 24.99±8.78^a^ | 72.00±16.87 | 91.218 | **0.001** |
| erucic acid (22:1 n-9) | 25 | 1.77±2.08 | 2.04±2.82 | 1.3 | 5.44±3.50^ab^ | 19.55±7.50 | 56.432 | **0.001** |
| behenic acid (C22:0) | 100 | 14.02±20.49 | 5.66±10.66 | 11.6 | 63.56±22.64^ab^ | 100.00±0.00 | 86.773 | **0.001** |
| nervonic acid C24:1 n-9 | 150 | 20.20±31.14 | 4.47±17.09^a^ | 60 | 88.09±24.70^ab^ | 150.00±0.00 | 98.249 | **0.001** |
| Wood tar acid (C24:0) | 100 | 12.68±17.51 | 5.53±11.92 | 37.8 | 57.55±21.87^ab^ | 100.00±0.00 | 90,748 | **0.001** |
| w3/w6 | 0.12 | 0.1111±0.0395 | 0.1461±0.0549^a^ | 1.26 | 0.1085±0.0344^b^ | 0.1375±0.0340 | 164.759 | **0.001** |
| Triene/tetraene | 0.01 | 0.2898±0.2105 | 0.5303±0.1998^a^ | 0.02 | 0.0183±0.0083^ab^ | 0.0175±0.005 | 58.2 | **0.001** |
| Total saturated fatty acid | 29908 | 4008.22±875.97 | 7093.00±2076.46^a^ | 5143 | 5866.49±1401.64^ab^ | 11813±2983 | 128.236 | **0.001** |
| Total monounsaturated fatty acid | 23265 | 2036.6±636.6 | 4684.2±1512.0^a^ | 1633 | 2643.5±950.7^ab^ | 9418.5±44251.4 | 153.5 | **0.001** |
| Total polyunsaturated fatty acid | 43038 | 5768.4±1002.6 | 9332.3±1828.4^a^ | 4816 | 7707.9±1642.7^ab^ | 17823±4151 | 208.684 | **0.001** |
| Total w3 | 4336 | 534.33±183.02 | 1082.29±436.13^a^ | 457 | 720.37±260.92^ab^ | 2017.75±489.06 | 83.467 | **0.001** |
| Total w6 | 37335 | 4956.6±905.3 | 7654.7±1656.0^a^ | 4138 | 6702.6±1481.1^ab^ | 15149±3766 | 185.487 | **0.001** |
| Total fatty acid | 96210 | 11792±1963 | 20940±4798^a^ | 11592 | 17363±10811^ab^ | 39055±9263 | 51.676 | **0.001** |
