# Supplementary material for: Fatty Acid Profiles and Their Association With Autoimmunity, Insulin Sensitivity and β Cell Function in Latent Autoimmune Diabetes in Adults
Source: Front Endocrinol (Lausanne). 2022 Jun 29;13:916981. doi: 10.3389/fendo.2022.916981 (PMC9276921; doi:10.3389/fendo.2022.916981)
Supplement: Supplementary file 1 [file DataSheet_1.zip › Supplementary Table 5.docx]

Supplementary Table 5. Clinical characteristics of different clusters of fatty acids

|  |  | Numbers of cluster | | | | |  |  |
| --- | --- | --- | --- | --- | --- | --- | --- | --- |
|  | 1 | 2 | 3 | 4 | 5 | 6 |  |  |
| n | 1 | **130** | **38** | 1 | **89** | 4 | *F* | *P* |
| age | 36 | **59.41±12.87** | **56.61±11.35** | 84 | **55.53±13.89^a^** | 41.25±17.95 | 2.456 | 0.088 |
| age at dx | 34 | **49.57±10.75** | **47.03±10.42** | 64 | **46.84±12.59** | 38.00±19.51 | 1.764 | 0.173 |
| Duration | 2 | **9.89±7.68** | **9.58±7.43** | 20 | **8.70±7.88** | 3.28±2.72 | 0.625 | 0.536 |
| BMI | 24.69 | **25.22±3.75** | **26.56±3.43** | 25.95 | **24.24±4.32^b^** | 24.69±2.73 | 4.798 | **0.009** |
| W | 95 | **90.43±12.92** | **94.68±9.00** | 103 | **88.93±11.60^b^** | 83.00±6.16 | 3.06 | **0.049** |
| SBP | 150 | **143.63±21.84** | **151.34±19.61** | 124 | **142.64±22.95^b^** | 124.75±14.29 | 2.274 | 0.105 |
| DBP | 94 | **79.88±11.13** | **85.13±12.67^a^** | 71 | **82.63±10.69** | 78.00±6.78 | 3.769 | **0.024** |
| Crea | 78.18 | **62.38±18.12** | **62.57±12.00** | 83.7 | **58.23±15.80** | 71.95±42.01 | 1.867 | 0.157 |
| eGFR | 90 | **87.86±7.49** | **89.23±3.49** | 90 | **88.72±4.87** | 81.26±17.49 | 0.949 | 0.389 |
| uAlb/Crea | 36.11 | **52.25±95.09** | **66.94±167.38** | 662.9 | **153.98±410.73** | 80.27±6.46 | 4.184 | **0.016** |
| UA |  | **303.85±93.74** | **349.93±90.09** |  | **312.99±86.63** |  | 3.78 | **0.024** |
| HbA1c | 13.5 | **8.19±1.79** | **8.92±2.36^a^** | 9.4 | **8.90±2.43^a^** | 11.60±1.08 | 3.668 | **0.027** |
| FBG | 15.9 | **8.55±3.25** | **9.28±3.23** | 9.12 | **9.11±3.86** | 13.31±3.08 | 1.021 | 0.362 |
| 1hBG | 10.08 | **13.30±3.96** | **13.96±4.51** | 13.85 | **13.63±4.34** | 17.94±2.68 | 0.416 | 0.66 |
| 2hBG | 5.76 | **14.10±5.27** | **14.74±5.99** | 19.92 | **14.77±5.37** | 18.83±4.16 | 0.473 | 0.623 |
| FCP（nmol/L) | 0.13 | **0.39±0.25** | **0.46±0.23** | 0.92 | **0.35±0.25** | 0.76±0.40 | 2.505 | 0.084 |
| 1hCP | 0.05 | **0.79±0.59** | **0.89±0.54** | 1.29 | **0.71±0.59** | 0.94±0.79 | 1.355 | 0.26 |
| 2hCP | 0.07 | **1.09±0.79** | **1.14±0.70** | 2.46 | **0.95±0.80** | 1.16±0.96 | 1.139 | 0.322 |
| log-ISI-CP | 3.52 | **2.89±0.48** | **2.71±0.22** | 2.31 | **3.00±0.60^ab^** | 2.46±0.21 | 4.333 | **0.014** |
| log-InsSecr-CP | -1.64 | **-0.86±0.52** | **-0.74±0.32** | -0.49 | **-1.01±0.64^ab^** | -0.84±0.34 | 3.667 | **0.027** |
| Tchol | 10.6 | **4.55±0.91** | **5.62±1.12^a^** | 2.89 | **5.47±1.30^a^** | 7.73±3.53 | 25.014 | **0.001** |
| Trig | 15.85 | **1.18±0.48** | **3.05±1.39^a^** | 1.94 | **1.83±0.98^ab^** | 14.38±13.67 | 71.028 | **0.001** |
| HDL-C | 1.01 | **1.26±0.78** | **1.18±0.30** | 0.79 | **1.29±0.37** | 1.04±0.51 | 1.641 | 0.196 |
| LDL-C | 1.74 | **2.44±0.60** | **2.87±0.83^a^** | 1.36 | **3.17±1.01^a^** | 1.95±0.82 | 22.073 | **0.001** |
