# Supplementary material for: Fatty Acid Profiles and Their Association With Autoimmunity, Insulin Sensitivity and β Cell Function in Latent Autoimmune Diabetes in Adults
Source: Front Endocrinol (Lausanne). 2022 Jun 29;13:916981. doi: 10.3389/fendo.2022.916981 (PMC9276921; doi:10.3389/fendo.2022.916981)
Supplement: Supplementary file 1 [file DataSheet_1.zip › Supplementary Table 6.docx]

Supplementary Table 6 Distribution of types of diabetes among the clusters of fatty acid profile

|  |  |  | Numbers of Cluster | | | | | Total |
| --- | --- | --- | --- | --- | --- | --- | --- | --- |
|  |  | 1 | 2 | 3 | 4 | 5 | 6 |  |
| type 1 | n | 1 | **26** | 0 | 0 | **36** | 0 | 63 |
|  | % | 1.6 | **28.3** | 0 | 0 | **57.1** | 0 | 100 |
| LADA | n | 0 | **13** | 0 | 1 | **30** | 2 | 46 |
|  | % | 0 | **28.30** | 0 | 2.2 | **65.2** | 4.3 | 100 |
| Type 2 | n | 0 | **90** | **38** | 1 | **19** | 2 | 149 |
|  | % | 0 | **60.4** | **25.5** | 0.4 | **12.8** | 1.3 | 100 |
| Total | n | 1 | **129** | **38** | 1 | **85** | 4 | 258 |
|  | % | 0.4 | **50** | **14.7** | 0.4 | **32.9** | 1.6 | 100 |
