# Supplementary material for: Fatty Acid Profiles and Their Association With Autoimmunity, Insulin Sensitivity and β Cell Function in Latent Autoimmune Diabetes in Adults
Source: Front Endocrinol (Lausanne). 2022 Jun 29;13:916981. doi: 10.3389/fendo.2022.916981 (PMC9276921; doi:10.3389/fendo.2022.916981)
Supplement: Supplementary file 1 [file DataSheet_1.zip › Supplementary Table 7.docx]

Supplementary Table 7 Spearman correlation analysis of diabetes-associated clinical parameters with fatty acid

| Fatty acid | Autoimmunity | | | Glycemic status | | | Insulin sensitivity & β cell function | | | |
| --- | --- | --- | --- | --- | --- | --- | --- | --- | --- | --- |
|  | GADA | ATG | ATPO | HbA1c | FBG | 2hBG | FCP | 2hCP | ISI | InScr |
| Kwai acid (C19:0) | **.198^**^** | **.134^*^** | **.134^*^** | 0.119 | 0.011 | 0.020 | **-.166^**^** | **-.182^**^** | **-.152^*^** | **-.167^**^** |
| Lauric acid (C12:0) | 0.118 | 0.005 | 0.021 | -0.103 | -0.099 | -0.063 | 0.050 | 0.119 | 0.068 | 0.116 |
| MLA (C14:1 n-5) | **.140^*^** | -0.029 | 0.056 | -0.004 | 0.102 | -0.032 | **.175^**^** | 0.062 | **.144^*^** | 0.055 |
| Myristic acid (C14:0) | **-.175^**^** | **-.130^*^** | **-.153^*^** | 0.087 | **.134^*^** | 0.083 | **.433^**^** | **.302^**^** | **.459^**^** | **.245^**^** |
| PLA (C16:1 n-7) | **-.315^**^** | **-.178^**^** | **-.219^**^** | 0.002 | **.128^*^** | 0.089 | **.453^**^** | **.323^**^** | **.464^**^** | **.275^**^** |
| Palmitic acid (C16:0) | 0.095 | -0.010 | 0.046 | **.237^**^** | **.175^**^** | **.152^*^** | **.150^*^** | 0.031 | **.190^**^** | -0.031 |
| ALA (C18:3 n-3) | **-.288^**^** | **-.146^*^** | **-.192^**^** | 0.054 | 0.085 | 0.074 | **.332^**^** | **.247^**^** | **.348^**^** | **.216^**^** |
| LIA (18:2 n-6) | -0.039 | -0.004 | 0.008 | **.228^**^** | **.165^**^** | 0.085 | 0.046 | -0.081 | 0.063 | -0.071 |
| Oleic acid (18:1 n-9) | **-.242^**^** | **-.177^**^** | **-.173^**^** | 0.111 | **.164^**^** | 0.075 | **.352^**^** | **.157^*^** | **.338^**^** | **.145^*^** |
| Stearic acid (18:0) | 0.111 | 0.042 | 0.088 | 0.053 | 0.035 | 0.027 | 0.084 | 0.042 | 0.072 | 0.041 |
| EPA (C20:5 n-3) | **-.397^**^** | **-.138^*^** | **-.303^**^** | 0.015 | 0.077 | -0.063 | **.158^*^** | **.151^*^** | **.159^**^** | **.187^**^** |
| AOA (C20:4 n-6) | **.362^**^** | 0.069 | 0.084 | **.132^*^** | 0.091 | 0.055 | -0.056 | **-.128^*^** | -0.046 | **-.124^*^** |
| ETA ( C20:3 n-6) | **-.397^**^** | -0.111 | **-.228^**^** | **-.126^*^** | 0.009 | 0.004 | **.488^**^** | **.397^**^** | **.468^**^** | **.390^**^** |
| EDA (C20:2 n-6) | **-.183^**^** | **-.128^*^** | **-.141^*^** | 0.086 | 0.110 | 0.073 | **.340^**^** | **.174^**^** | **.339^**^** | **.162^**^** |
| ENA (C20:1 n-9) | **-.175^**^** | **-.145^*^** | -0.083 | 0.091 | 0.116 | 0.029 | .**244^**^** | 0.074 | **.213^**^** | 0.087 |
| AIA (C20:0) | **.602^**^** | **.141^*^** | **.267^**^** | 0.112 | -0.020 | 0.005 | **-.139^*^** | **-.169^**^** | **-.150^*^** | **-.167^**^** |
| DHA (C22:6 n-3) | 0.022 | -0.014 | -0.109 | 0.082 | 0.093 | 0.005 | 0.103 | 0.047 | 0.107 | 0.071 |
| DPA (C22:5 n-3) | **-.414^**^** | **-.187^**^** | **-.233^**^** | 0.078 | **.165^**^** | 0.012 | **.281^**^** | **.140^*^** | **.273^**^** | **.150^*^** |
| DTA (C22:4 n-6) | **.289^**^** | 0.006 | **.128^*^** | **.171^**^** | **.128^*^** | **.149^*^** | 0.110 | -0.030 | **.131^*^** | -0.073 |
| ECA (C22:1 n-9) | **.557^**^** | 0.112 | **.227^**^** | 0.039 | -0.006 | -0.022 | -0.106 | **-.140^*^** | **-.124^*^** | **-.132^*^** |
| Behenic acid (C22:0) | **.633^**^** | **.134^*^** | **.293^**^** | **.125^*^** | 0.017 | 0.029 | **-.186^**^** | **-.210^**^** | **-.195^**^** | **-.210^**^** |
| NNA (C24:1 n-9) | **.636^**^** | **.139^*^** | **.283^**^** | 0.110 | -0.003 | 0.038 | **-.157^*^** | **-.177^**^** | **-.161^**^** | **-.174^**^** |
| WTA (C24:0) | **.632^**^** | 0.118 | **.289^**^** | **.121^*^** | 0.026 | 0.041 | **-.172^**^** | **-.205^**^** | **-.182^**^** | **-.205^**^** |
| Triene/tetraene | **-.656^**^** | -.186^**^ | **-.277^**^** | **-.134^*^** | 0.002 | -0.036 | **.357^**^** | **.325^**^** | **.344^**^** | **.319^**^** |
| n-3/n-6 | **-.282^**^** | -.130^*^ | **-.280^**^** | -0.096 | -0.005 | -0.068 | **.241^**^** | **.266^**^** | **.233^**^** | **.285^**^** |
| n-3 | **-.201^**^** | -0.076 | **-.197^**^** | 0.068 | 0.117 | 0.017 | **.203^**^** | **.149^*^** | **.214^**^** | **.161^**^** |
| n-6 | 0.067 | 0.017 | 0.027 | **.246^**^** | **.173^**^** | 0.088 | 0.022 | -0.116 | 0.041 | -0.108 |
| MUFA | **-.218^**^** | **-.166^**^** | **-.161^**^** | 0.104 | **.160^**^** | 0.078 | **.352^**^** | **.164^**^** | **.340^**^** | **.148^*^** |
| PUFA | 0.003 | -0.003 | -0.017 | .227^**^ | **.175^**^** | 0.086 | 0.081 | -0.057 | 0.102 | -0.052 |
| SFA | 0.095 | -0.011 | 0.050 | **.203^**^** | **.137^*^** | 0.107 | **.148^*^** | 0.039 | **.171^**^** | -0.003 |
| TFA | 0.007 | -0.049 | -0.030 | **.203^**^** | **.153^*^** | 0.100 | **.196^**^** | 0.058 | **.215^**^** | 0.033 |

*P<0.05, **P<0.001; Degree of freedom=221. ETA: Eicostrienoic acid, EDA: Eicosadienoic acid, ESA: Eicosenoic acid, ALA: Linolenic acid, MUFA: monounsaturated fatty acid, DPA: docosapentaenoic Acid, SFA: total saturated fatty acid, WTA: wood tar acid, AIA: Arachic acid, ECA: Erucic Acid, AOA: Arachidonic Acid, EPA: Eicosapentaenoic Acid, DHA: Docosahexaenoic Acid, PUFA: polyunsaturated fatty acid, TFA: total fatty acid, LIA: Linoleic Acid.
